# A novel preoperative simulation approach for pediatric kidney transplantation to support donor-recipient size matching using dialysis fluid-enhanced CT imaging and 3D printing

**DOI:** 10.3389/frtra.2026.1861599

**Published:** 2026-06-22

**Authors:** Julia Bojstedt, Lena Gordon Murkes, Vasileios Tsirkos, Johan Nordström

**Affiliations:** 1 Division of Perioperative Medicine and Intensive Care (PMI), Karolinska University Hospital, Stockholm, Sweden; 2 3D Center, Karolinska University Hospital, Stockholm, Sweden; 3 Department of Pediatric Radiology, Karolinska University Hospital, Stockholm, Sweden; 4 Division of Transplantation Surgery, Department of Clinical Science, Intervention and Technology (CLINTEC), Karolinska Institute, Stockholm, Sweden; 5 Department of Transplantation Surgery, Karolinska University Hospital Huddinge, Stockholm, Sweden

**Keywords:** computed tomography, donor-recipient mismatches, graft accommodation, image segmentation, living-donor kidney transplantation, pediatric kidney transplantation, three-dimensional printing (3D printing), virtual surgical planning (VSP)

## Abstract

**Background:**

Size mismatch between adult donor kidneys and small pediatric recipients remains a significant technical challenge in pediatric kidney transplantation. To address this, we explored a novel preoperative method to evaluate donor-recipient anatomical compatibility.

**Methods:**

Three pediatric recipients underwent pre-transplant assessments using segmented CT data from living donors to generate patient-specific 3D-printed kidney models. Digital simulation was performed by virtually positioning the donor kidney within fluid-enhanced recipient CT datasets acquired following intraperitoneal dialysis fluid administration. It was hypothesized that this combined approach would enable objective preoperative assessment of spatial fit and surgical feasibility.

**Results:**

The preoperative simulation and modeling approach contributed to successful kidney transplantation in all three children without intraoperative complications attributable to graft placement. Postoperative recovery was uneventful, and graft function remained satisfactory at 6-month follow-up.

**Conclusion:**

The integration of 3D-printed donor kidneys with digital simulation based on dialysis fluid-enhanced CT imaging offers a feasible approach for preoperative donor-recipient size matching in small pediatric recipients. This method may enhance surgical planning, allow safer use of living-donor organs in very small children, and reduce time spent on dialysis.

## Introduction

Kidney transplantation is the preferred treatment for children with end-stage renal disease because of superior long-term outcomes with respect to growth, neurodevelopment, quality of life, and survival compared with dialysis (1–3). Pediatric recipients present unique anatomical and physiological challenges, particularly when donor kidneys originate from adult living donors and thus exceed the available intra-abdominal space of the recipient. Size mismatch between adult donor kidneys and small children is a well-recognized hurdle, complicating graft placement, vascular anastomoses, and graft perfusion dynamics (4–6). Although the exact frequency of failure directly attributable to donor-recipient size mismatch is not consistently reported, its clinical consequences are reflected in early surgical complications, particularly vascular thrombosis, graft hypoperfusion, difficulty with abdominal wall closure, and, in selected cases, renal allograft compartment syndrome (4, 7–9). In young pediatric recipients, early urological or vascular complications have been reported in approximately one quarter of cases and are associated with reduced graft survival (8). Contemporary reviews also report renal thrombosis in 3%–12% and arterial stenosis in 3%–15% of pediatric kidney transplant recipients, underscoring the importance of anticipating mechanical and vascular constraints before implantation (9). To date, preoperative assessment has largely relied on standard imaging such as ultrasound and computed tomography (CT) or magnetic resonance imaging, anthropometric donor-recipient indices including weight ratio and body surface area ratio, and the surgeon's subjective judgment.

Objective tools for simulation of graft placement and space occupancy within the recipient's peritoneal cavity are still limited. At the same time, three-dimensional (3D) printing, augmented reality, and artificial intelligence modalities are increasingly applied to surgical planning, offering patient-specific anatomical models, tactile preoperative rehearsal, and immersive virtual simulation (10–12). In this context, we introduced a novel method combining 3D-printed donor kidney models with dialysis fluid-enhanced CT of the pediatric recipient's peritoneal cavity to digitally and physically simulate donor-recipient fit and anticipated graft placement. The objective was to evaluate the feasibility of this method and report early outcomes in a small case series.

## Materials and equipment

### Donor assessment with virtual and physical model generation

Living donors underwent standard preoperative imaging, including multiphasic contrast-enhanced CT for detailed assessment of renal vascular and parenchymal anatomy.

Multiphase contrast-enhanced CT imaging of the living donor kidneys included arterial, venous, and excretory phases to enable visualization of the renal artery, renal vein, and ureter, respectively. For the purposes of this study, donor CT datasets were required to include thin-slice reconstructions of 0.6 mm in the arterial, venous, and excretory phases. Although donor CT examinations were performed at different hospitals and acquisition parameters therefore followed local clinical routines, the imaging protocols were sufficiently comparable in phase timing, anatomical coverage, and spatial resolution to permit segmentation and use in the study workflow. CT scanner type, acquisition settings, and radiation dose were not standardized across patients and were therefore not included in the analysis.

Donor kidney segmentation was performed using dedicated medical imaging software (Mimics Medical Core, Materialise, Leuven, Belgium). Threshold-based segmentation was applied to each contrast phase separately, exploiting phase-specific differences in attenuation values to isolate the relevant anatomical structures. The arterial, venous, and excretory phase datasets were inherently co-registered as part of the multiphasic CT acquisition, allowing direct segmentation of the renal parenchyma and arterial anatomy from the arterial phase, the renal vein from the venous phase, and the ureter from the excretory phase. The resulting segmentations were combined within the same spatial reference frame to generate a comprehensive 3D model of the donor kidney and associated vascular and urinary structures.

Manual refinement of the segmentation masks, including artifact removal and correction of anatomical boundaries, was performed as needed to improve anatomical delineation. No wrapping or object operations intended to alter kidney morphology were applied. The donor kidney 3D volumes reported in Table 1 were calculated in Mimics from the final segmentation masks before export of the virtual surface models. Limited surface smoothing was applied only to the exported 3D objects used for visualization and 3D printing. The final models were reviewed by a radiologist and the transplant surgeon to confirm anatomical correctness and suitability for study purposes.

**Table 1:** Donor kidney characteristics.

Donor	Donated side	A/V/U n^a^	Kidney dimension (W × D × H), cm	Estimated volume, cm^3^^b^	3D volume, cm^3^^c^	Split renal function (Right/Left), %
1	Left	2/1/1	5.1 × 4.4 × 10.8	126.0	140.0	50/50
2	Left	1/1/1	4.9 × 4.7 × 11.3	135.3	148.8	52/48
3	Right	1/1/1	3.8 × 5.4 × 9.8	104.6	111.7	43/57

A, artery; V, vein; U, ureter.

a n = number of renal arteries, renal veins, and ureters identified on donor CT and included in the segmented donor kidney model.

b Estimated volume calculated using an ellipsoid approximation (π/6 × W × D × H).

c 3D volume generated from virtual surface model, based on segmentation of donor CT data.

**Figure 1:**
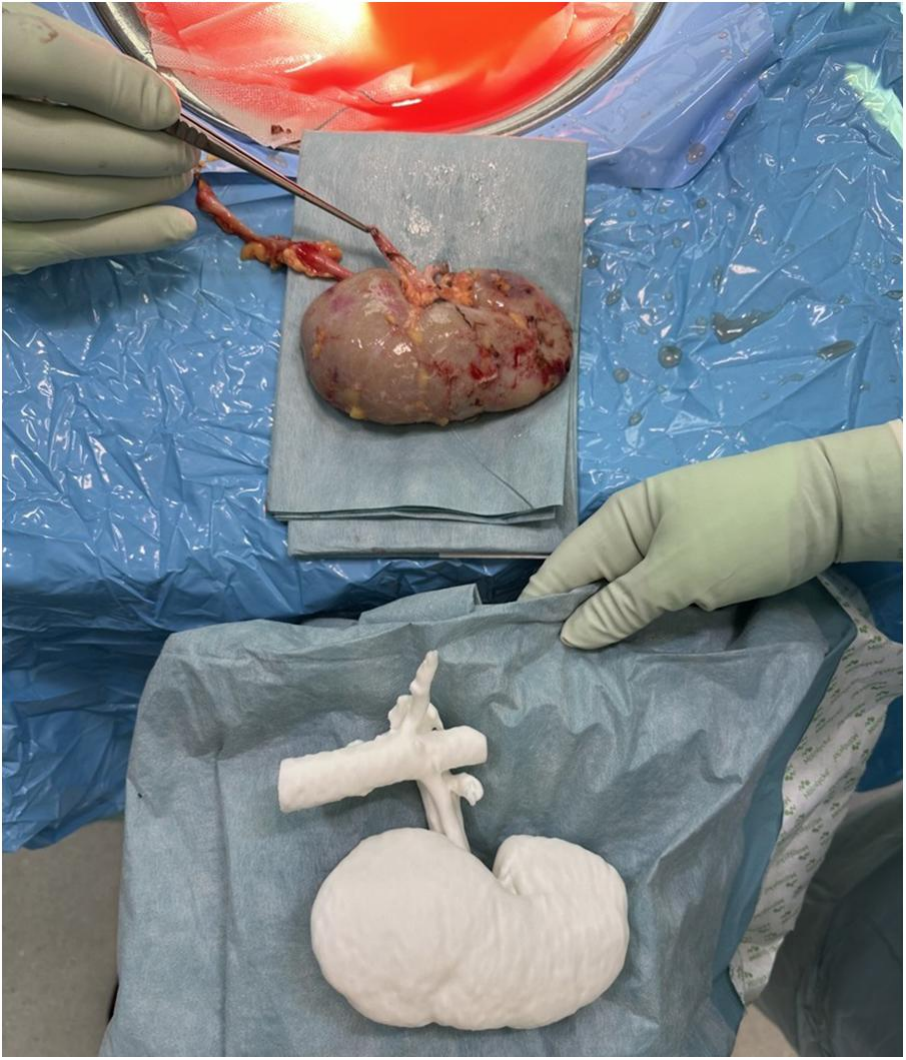
Intraoperative comparison of the living-donor kidney graft and its patient-specific 3D-printed replica. The explanted donor kidney is shown on the back table, alongside the corresponding true-to-scale 3D-printed model.

In addition, a physical 3D model of each donor kidney was printed at true scale using a desktop 3D printer (Bambu Lab X1C, Bambu Lab, Shenzhen, China) and polylactic acid (PLA) material (Figure 1). The physical model enabled tactile assessment of renal anatomy and was used to corroborate graft dimensions, orientation, and spatial configuration during preoperative planning, thereby supporting appraisal of anticipated graft accommodation.

### Recipient assessment and virtual model generation

Recipient imaging was performed using non-contrast abdominal CT with intraperitoneal dialysis fluid serving as a contrast surrogate. This fluid-filled abdomen technique was used to delineate the peritoneal cavity and available intra-abdominal space for virtual surgical planning. Each recipient underwent CT under sedation with dexmedetomidine hydrochloride (Dexdor). Intraperitoneal dialysis fluid was instilled via the existing peritoneal dialysis catheter in a volume corresponding to the patient's usual nocturnal dialysis prescription, equivalent to approximately 30 mL/kg. No additional contrast agent was administered, as the attenuation difference between dialysis fluid and surrounding soft tissue was sufficient for anatomical delineation.

The technique enabled controlled distension of the peritoneal cavity with a clinically tolerated fluid volume without compromising intra-abdominal pressure. Although donor and recipient CT protocols differed, this did not affect dimensional assessment, spatial orientation, or the feasibility of 3D planning and measurements. Recipient CT datasets were subsequently used for virtual model generation and assessment of the available intra-abdominal space for anticipated graft accommodation.

A dedicated pediatric non-contrast CT protocol was used on a dual-source CT scanner (SOMATOM Drive, Siemens Healthineers, Forchheim, Germany). Scan protocol parameters: 80 kV, (dualsource protocol) pitch 2.5, nominal collimation 38.4 mm, rotation time 0.28 s, and automated tube current modulation. Z-axis coverage extended from the xiphoid process to the symphysis pubis. The median CTDIvol was 0.9 mGy.

The abdominal CT datasets provided a 3D volumetric depiction of the peritoneal cavity, delineating available intraperitoneal space, including dependent fluid-filled compartments. Peritoneal cavity volume was quantified, and anatomic constraints were characterized by measuring clearance distances to key structures, including the urinary bladder, diaphragm, and pelvic floor.

CT datasets were segmented to generate 3D models of the recipient anatomy, including the liver, abdominal wall (skin), aortic bifurcation, spine, and rib cage, using dedicated medical imaging software (Mimics Medical Core, Materialise, Leuven, Belgium). Segmentation was primarily performed using threshold-based techniques, which exploit differences in tissue attenuation values expressed in Hounsfield units (HU). In this approach, HU ranges were selected to isolate specific anatomical structures based on their characteristic density (e.g., bone, soft tissue, or fluid). Voxels with attenuation values within the selected threshold range were automatically classified as part of the target structure, forming an initial segmentation mask. These masks were subsequently refined using manual editing and region-growing tools to ensure accurate delineation of anatomical boundaries and to correct for partial volume effects or overlapping attenuation values.

### Virtual and physical donor-recipient fit simulation

Donor kidney virtual surface models derived from segmented donor CT datasets were imported into dedicated medical imaging software (Mimics Medical Core and Mimics Viewer, Materialise, Leuven, Belgium) and manually aligned with each recipient CT dataset. Recipient abdominal CT had been acquired after intraperitoneal instillation of dialysis fluid via the peritoneal dialysis catheter to delineate the intraperitoneal space. The segmented adult living-donor kidney model was then virtually positioned within the recipient abdomen to support preoperative size matching and assessment of anticipated graft accommodation.

**Figure 2:**
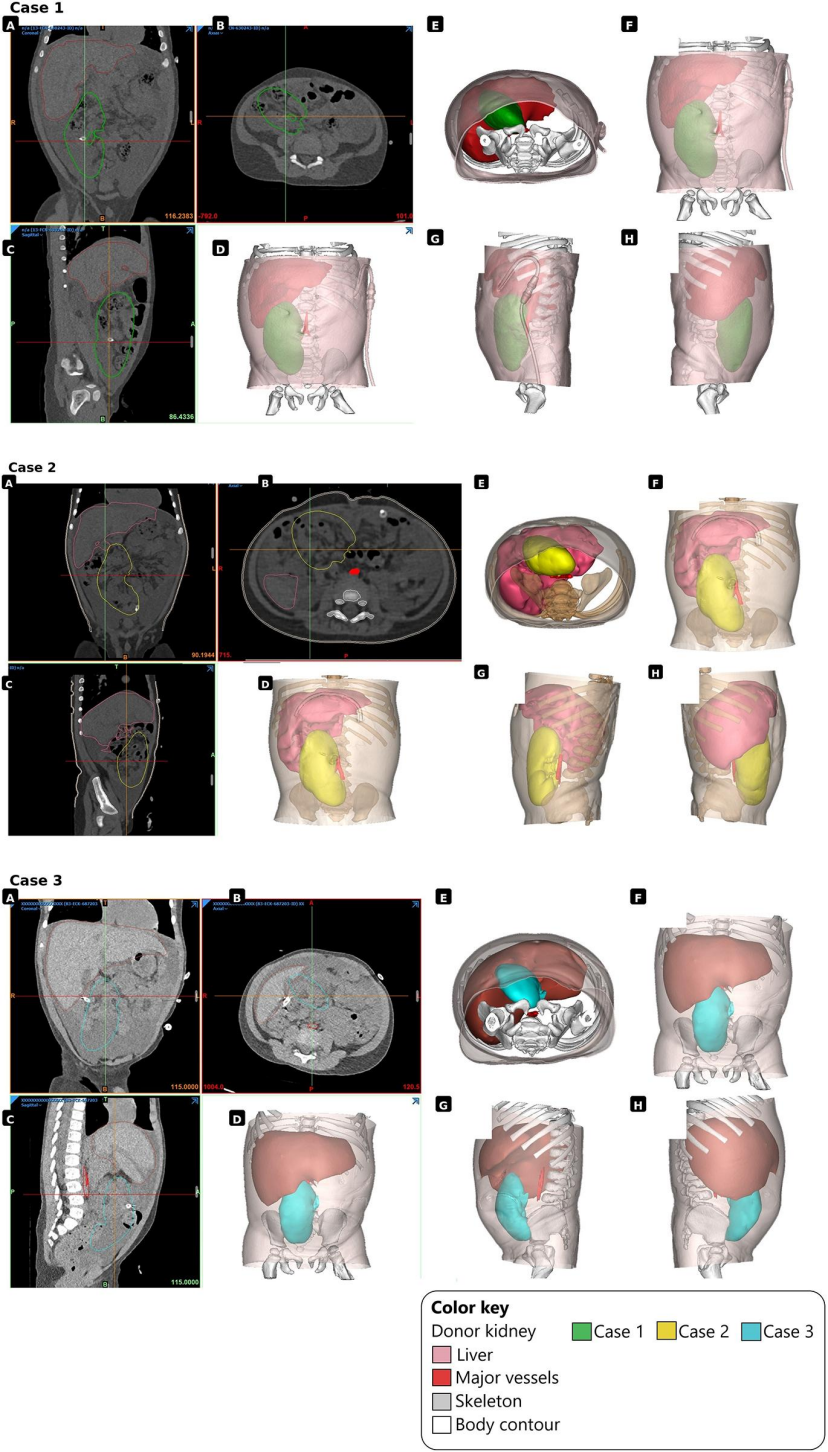
Dialysis fluid-enhanced CT and virtual donor-recipient fit simulation in three pediatric recipients. For each case, abdominal CT was acquired after intraperitoneal instillation of dialysis fluid via the peritoneal dialysis catheter to expand the intraperitoneal space. A segmented adult living-donor kidney model was virtually positioned within each recipient dataset to support preoperative size matching and assessment of anticipated graft accommodation. Within each case, panels (A–C) show coronal, axial, and sagittal CT planes with the virtually positioned donor kidney contour. Panel **(D)** shows a 3D overview of the planned graft position within the recipient body contour. Panels **(E–H)** provide complementary 3D viewpoints to illustrate graft orientation, clearance to the abdominal wall, and relationships to adjacent structures. Color coding is provided in the in-figure key.

Rigid transformations allowed controlled 3D rotation and translation of the donor kidney model within the recipient dataset. Virtual placement was reviewed in coronal, axial, and sagittal planes, including assessment of the donor kidney contours in relation to recipient anatomy, as well as in 3D renderings. The simulation was used to evaluate anatomical compatibility, anticipated graft orientation, clearance to the abdominal wall, and potential sites of mechanical constraint related to limited intraperitoneal space (Figure 2).

**Figure 3:**
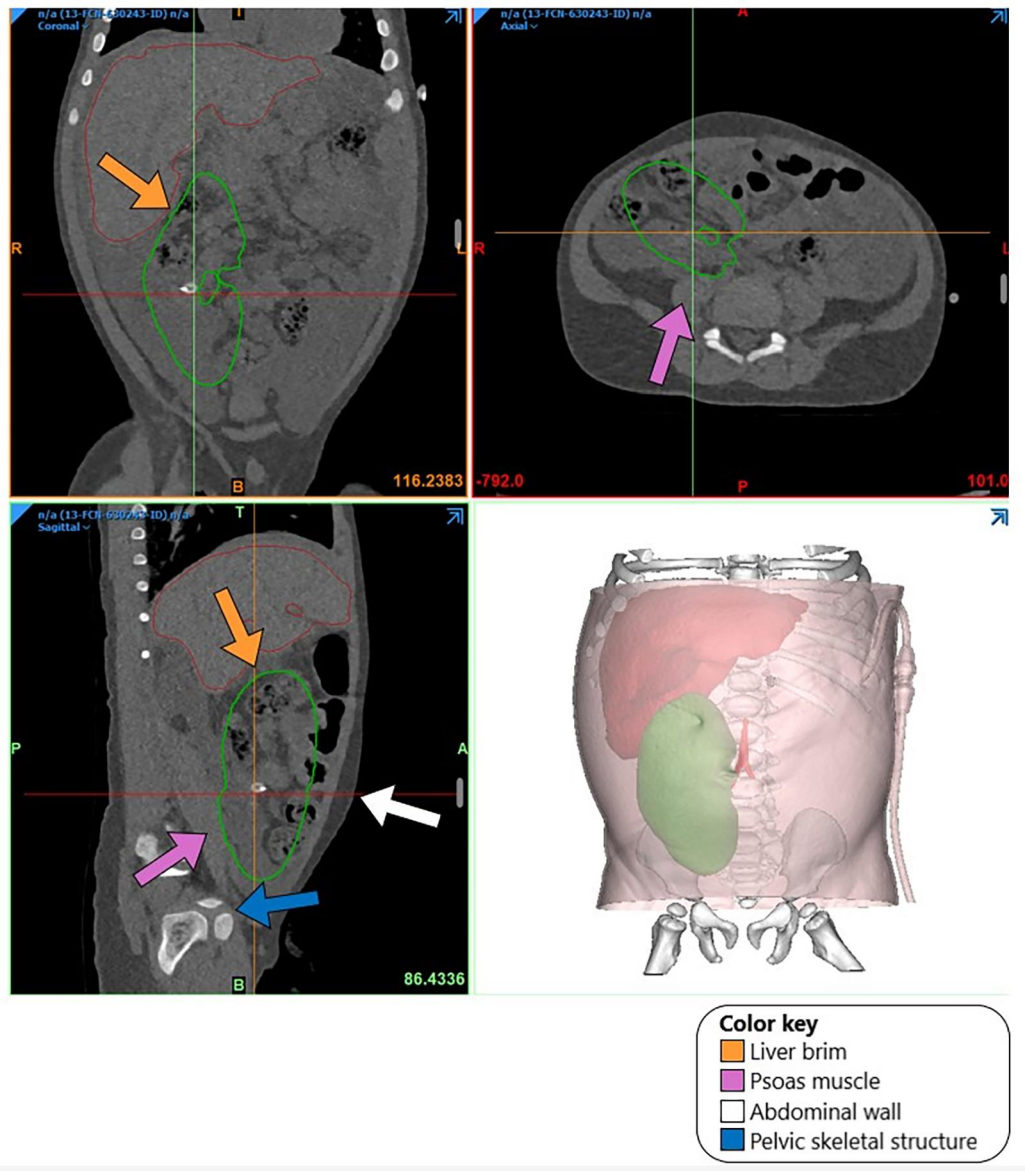
Relevant anatomical structures considered during operative planning. The donor kidney contour is shown in green in relation to relevant recipient structures. Arrows indicate the liver margin, psoas muscle, abdominal wall, and pelvic skeletal structures, as defined in the color key.

Relevant anatomical constraints included the pelvic skeletal structures, psoas muscle, liver, and abdominal wall. Figure 3 shows one representative example from the three recipients included in the study. Intestines were not considered fixed constraints because of their mobility within the abdominal cavity. The virtual simulations were assessed qualitatively with regard to overall graft dimensions, anticipated orientation, spatial accommodation, clearance to the abdominal wall, and relationship to recipient vasculature and adjacent organs. The transplant surgeon's assessment of the virtual and digital presentations was essential for interpreting surgical feasibility, anticipated graft positioning, and potential intraoperative constraints. No predefined millimeter-based thresholds or exact distance measurements were applied. Potential conflict zones, including anticipated vascular kinking and graft compression during abdominal wall closure, were identified and incorporated into the operative plan.

In parallel, true-scale 3D-printed donor kidney models were produced as visual and tactile aids. During outpatient visits, the models were placed externally on the recipient's abdomen to illustrate graft size and anticipated accommodation. They were also used during multidisciplinary preoperative planning and to support communication with the families, but not for direct physical fit testing or formal quantitative measurement.

## Methods

### Study design and patients

**Figure 4:**
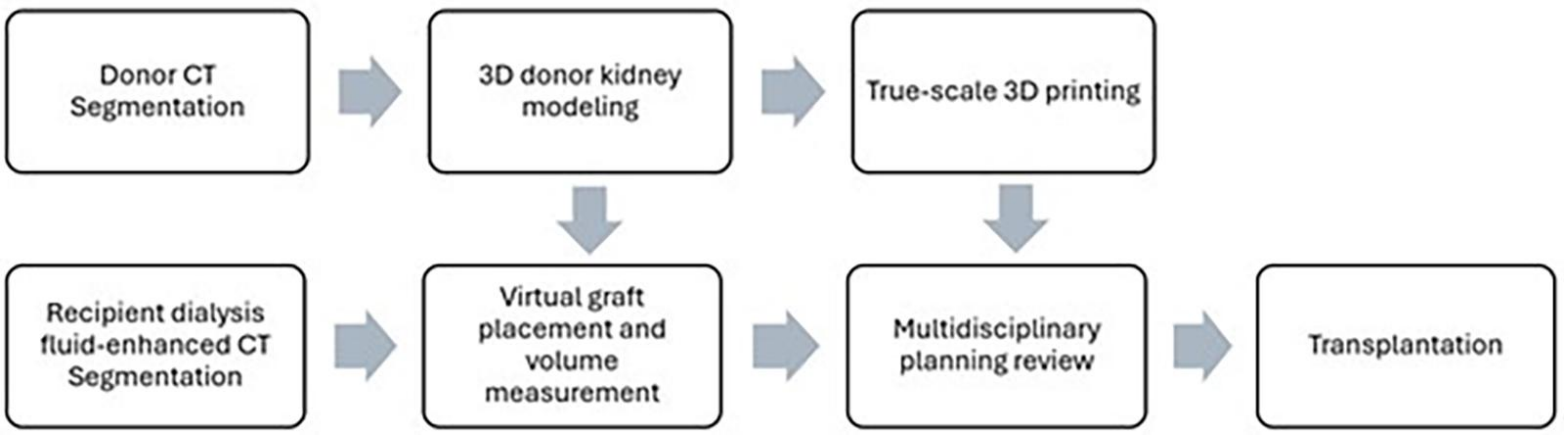
Workflow overview.

A retrospective case series was conducted including three pediatric patients who underwent pre-transplant evaluation and kidney transplantation at our institution between 2024 and 2025. All patients were scheduled to receive living-donor kidney transplants from adult donors and underwent preoperative donor-recipient fit simulation using the method described. The workflow is shown in Figure 4. The study was approved by the Swedish Ethical Review Authority (Dnr 2025-07189-02).

### Transplantation and follow-up

Recipients underwent standard kidney transplantation through a midline laparotomy with intraperitoneal placement of the donor kidney. Arterial anastomosis was performed to the distal abdominal aorta, venous anastomosis to the distal inferior vena cava, and ureteroneocystostomy to the bladder. All recipients received protocol-based maintenance immunosuppression with tacrolimus, mycophenolate mofetil, and corticosteroids. Pneumocystis jirovecii pneumonia prophylaxis was administered for 6 months, and cytomegalovirus prophylaxis for 3 months, in accordance with local standard of care at our center.

The intra-operative course was monitored for any technical difficulties related to graft positioning or abdominal wall closure. Post-operative monitoring included graft function measured as serum creatinine at 1, 3, and 6 months. For this pediatric patient population, the serum creatinine reference range is <41 µmol/L. Graft function was also measured as adjusted estimated glomerular filtration rate (eGFR) using the Schwartz formula (13). Normal or high eGFR is ≥90 mL/min/1.73m^2^ according to KDIGO (14). Complications were recorded using the Clavien–Dindo classification (15, 16). The primary outcome was graft survival at 6 months. Secondary outcomes included graft function, perioperative complications related to limited intra-abdominal space and graft fit, abdominal compartment syndrome, and wound closure complications.

## Results

### Recipient and donor characteristics

We included three pediatric recipients. Table 2 summarizes recipient characteristics. The first patient had bilateral renal dysplasia with vesicoureteral reflux and initiated peritoneal dialysis at one month of age, remaining on peritoneal dialysis for 23 months prior to transplantation. The second patient had bilateral renal dysplasia and started peritoneal dialysis at two weeks of age and remained on peritoneal dialysis for 27 months before transplantation. The third patient was healthy during the first year of life but developed pneumococcal-associated hemolytic uremic syndrome at 18 months of age, resulting in kidney failure. Peritoneal dialysis was initiated and continued for 18 months prior to transplantation.

**Table 2:** Recipient characteristics.

Recipient	Age (months)	Weight (kilograms)	Height (centimeters)	Time on PD (months)
1	23	11.5	84	23
2	27	12.7	84	27
3	33	13.6	87	18
Mean (range)	27.7 (23–33)	12.6 (11.5–13.6)	85 (84–87)	22.7 (18–27)

Values are presented as individual recipient values and mean (range). PD, peritoneal dialysis.

Donor and donor kidney characteristics are presented in Tables 1, 3.

**Table 3:** Donor characteristics.

Donor	Age (years)	Sex (F/M)	Weight (kilograms)	Height (centimeters)	Body mass index (kg/m^2^)
1	42	M	57.7	168	20.4
2	43	F	73	159	28.9
3	32	F	72	162	27.4
Mean (range)	39 (32–43)		67.6 (57.7–73)	163 (159–168)	25.6 (20.4–28.9)

Values are presented as individual donor values and mean (range). In the summary row, sex is presented as female/male count. F, female; M, male; BMI, body mass index.

### Operative and short-term outcomes

All three transplantations were completed without intraoperative complications related to graft placement, graft accommodation, or abdominal wall closure. The early postoperative course was uncomplicated, with no episodes of abdominal compartment syndrome or wound closure complications. At 6-month follow-up, graft survival was 100 percent, and none of the recipients required dialysis reinstitution. Secondary outcomes were favorable, with good graft function at 6 months, including a mean serum creatinine of 35.0 µmol/L (Table 4) and a mean eGFR of 94.4 mL/min/1.73 m^2^ (Table 5). No perioperative complications attributable to limited intra-abdominal space or graft fit were observed. Mean length of hospital stay was 15 days, with a range of 13–17 days.

**Table 4:** Serum creatinine before and after transplantation in recipients.

	Serum creatinine (µmol/L)			Mean (range)
Time point	Recipient 1	Recipient 2	Recipient 3	All patients
Pretransplant	550	527	637	571.3 (527–637)
Post-transplant day 1	21	12	47	26.7 (12–47)
Post-transplant week 1	14	9	14	12.3 (9–14)
Post-transplant month 1	22	19	19	20 (19–22)
Post-transplant month 2	28	41	20	29.7 (20–41)
Post-transplant month 3	30	37	26	31 (26–37)
Post-transplant month 6	35	36	34	35.0 (34–36)

Values are presented in µmol/L for individual recipients and as mean (range). The local upper reference limit for serum creatinine in this pediatric age group was <41 µmol/L.

**Table 5:** eGFR before and after transplantation in recipients.

Time point	Recipient 1 height (cm)	eGFR	Recipient 2 height (cm)	eGFR	Recipient 3 height (cm)	eGFR	Mean eGFR (range)
Pretransplant	84	5.6	84	5.8	87	5.0	5.5 (5.0–5.8)
Post-transplant day 1	84	146.0	84	255.5	87	67.6	156.4 (67.6–255.5)
Post-transplant week 1	84	219.0	84	340.7	87	226.8	262.2 (219.0–340.7)
Post-transplant month 1	84	139.4	84	161.4	87	167.1	156.0 (139.4–167.1)
Post-op month 2	84	109.5	84	74.8	87	158.8	114.4 (74.8–158.8)
Post-transplant month 3	85.5	104.0	85.6	84.4	89.4	125.5	104.7 (84.4–125.5)
Post-transplant month 6	88	91.8	91.2	92.5	92.3	99.1	94.4 (91.8–99.1)

eGFR is presented as mL/min/1.73 m^2^ and was calculated using the bedside Schwartz formula: eGFR = 0.413 × height (cm)/serum creatinine (mg/dL), equivalent to 36.5 × height (cm)/serum creatinine (µmol/L). Heights used for calculation at each time point are reported. Values are presented for individual recipients and as mean (range). Early post-transplant eGFR values, particularly on day 1 and week 1, should be interpreted with caution because serum creatinine may not have reached steady state and very low creatinine values may overestimate eGFR. An eGFR ≥90 mL/min/1.73 m^2^ is generally considered within the normal range.

Postoperative complications were recorded from transplantation to 6 months post-transplantation and graded according to the Clavien–Dindo classification, as summarized in Table 6. A total of 12 complication events were observed, including 6 during the index admission and 6 after discharge. Most events were grade II (9 events), and one grade IIIb complication occurred; no grade IV or V events were recorded. No complications were attributable to donor-recipient size mismatch or limited intra-abdominal space, and no events related to graft accommodation, abdominal compartment syndrome, or abdominal wall closure were observed.

**Table 6:** Postoperative complications by timing and Clavien–Dindo grade.

Time point	Complication	Management	Clavien–Dindo grade	Number of events
In-hospital	Non-infectious diarrhea	Conservative management	I	1
In-hospital	Need for nutritional support	Total parenteral nutrition	II	1
In-hospital	Infectious diarrhea	Antibiotic therapy	II	2
In-hospital	Respiratory tract infection	Antibiotic therapy	II	1
In-hospital	Lower urinary tract intervention	Cystoscopy under general anesthesia	IIIb	1
Post-discharge	Non-infectious diarrhea	Conservative management	I	1
Post-discharge	Urinary tract infection	Antibiotic therapy	II	1
Post-discharge	CMV infection	Antiviral therapy	II	1
Post-discharge	BK viremia	Reduction of immunosuppression, no antiviral therapy	II	2
Post-discharge	EBV viremia	Reduction of immunosuppression, no antiviral therapy	II	1

Values represent complication events, not number of affected recipients. Clavien–Dindo grade II included events requiring pharmacological treatment, antiviral or antibiotic therapy, nutritional support, or adjustment of immunosuppression. Grade IIIb included intervention under general anesthesia. CMV, cytomegalovirus; BK, BK polyomavirus; EBV, Epstein–Barr virus.

## Discussion

In this small case series, we demonstrate the feasibility of integrating donor kidney segmentation, virtual donor-recipient fit simulation, true-scale 3D-printed donor kidney models, and dialysis fluid-enhanced CT of the recipient abdomen to support preoperative planning in pediatric kidney transplantation. The method was developed to address the specific challenge of implanting adult living-donor kidneys in small pediatric recipients, where limited intraperitoneal space may increase the risk of difficult graft positioning, vascular kinking, graft compression, impaired perfusion, or renal allograft compartment syndrome (4–6). By combining donor-specific and recipient-specific imaging, the workflow provided a patient-specific assessment of anticipated graft accommodation beyond conventional imaging, anthropometric indices, and surgeon experience.

The virtual simulation was primarily used as a qualitative surgical planning tool. The donor kidney model was positioned at true scale within the recipient CT dataset and evaluated in multiplanar views and 3D renderings. Assessment focused on overall graft size, anticipated orientation, spatial accommodation, clearance to the abdominal wall, and the relationship to relevant anatomical constraints, including the pelvic skeletal structures, psoas muscle, liver, abdominal wall, and recipient vasculature. No predefined millimeter-based clearance thresholds were applied. Surgical feasibility was instead interpreted as a composite assessment of graft orientation, vascular reach, avoidance of compression, expected abdominal wall closure, and the transplant surgeon's evaluation of potential intraoperative constraints. This is important because spatial suitability in small pediatric recipients is unlikely to be captured by a single distance measurement.

The difference between ellipsoid-based volume estimates and CT-derived 3D segmented volumes is also relevant. The ellipsoid approximation is a well-established and commonly used radiological method for estimating organ volume, including renal volume, and provides a practical descriptive estimate based on maximal length, width, and depth. However, because the kidney has an irregular 3D shape, CT-derived segmentation is likely to provide a more anatomically representative estimate of graft morphology and volume. In the present study, the ellipsoid approximation was included only as a descriptive measure and was not used for virtual placement, proximity assessment, or surgical decision-making. The donor-recipient fit assessment was based on the true-scale segmented donor kidney model positioned within the recipient CT dataset, together with the surgeon's evaluation of orientation, accommodation, and potential mechanical constraints.

The physical 3D-printed kidney models had a complementary role. They provided visual and tactile information on graft size and morphology during multidisciplinary planning and communication with families. During outpatient visits, the models were placed externally on the recipient's abdomen to illustrate graft size and anticipated accommodation. However, they were not used for direct physical fit testing, formal quantitative measurement, or prediction of intra-abdominal accommodation. Because the models were printed from rigid material, they did not reproduce soft-tissue deformability, abdominal wall compliance, or intraoperative tissue handling; nor were they intended to serve this function. Future developments using flexible or tissue-specific materials may improve the ability to simulate compression, organ displacement, and abdominal wall closure.

Reports describing patient-specific 3D printing and virtual graft accommodation assessment in pediatric kidney transplantation remain scarce. Outside transplantation, a growing body of evidence supports patient-specific 3D printing and extended reality planning as tools to improve spatial understanding and surgical planning. Systematic reviews and meta-analyses in orthopedic trauma suggest that 3D-printed models may reduce operative time and improve intraoperative orientation, and some analyses report lower complication rates compared with conventional planning (17–19). Similarly, augmented, mixed, and virtual reality platforms have been associated with improved spatial understanding and more efficient preoperative planning workflows (20–22). The present workflow therefore contributes to the emerging field of technology-assisted planning in complex transplantation.

Several limitations warrant consideration. The sample size was small, reflecting the limited number of small pediatric recipients undergoing living-donor kidney transplantation during the study period. The retrospective design limits generalizability and precludes assessment of comparative effectiveness vs. standard planning alone. Postoperative CT was not performed because it is not part of routine clinical follow-up and would have added radiation exposure without clinical indication. Therefore, exact postoperative graft position and distance from the abdominal wall could not be validated radiologically. In this study, concordance between the preoperative simulation and clinical reality was instead supported by intraoperative feasibility, uncomplicated abdominal closure, preserved graft perfusion, and favorable early postoperative outcomes. In addition, the printed models were not independently validated by post-manufacturing CT or volumetric comparison with the digital model. The simulation also focused on geometric fit and did not account for dynamic factors such as postoperative edema, abdominal wall compliance, respiratory motion, organ displacement, patient positioning, or hemodynamic consequences of implanting an adult kidney in a small child.

The potential applications of this workflow may extend beyond adult-to-pediatric kidney transplantation. Patient-specific virtual planning could be useful in recipients with congenital malformations, abnormal pelvic or abdominal anatomy, prior extensive abdominal surgery, complex vascular anatomy, re-transplantation, or other anatomical variants where standard imaging does not fully answer the surgical feasibility question. Similar principles may also be applicable to other complex abdominal transplant procedures in which organ size, orientation, vascular access, and spatial constraints are central to operative planning.

In conclusion, this case series demonstrates the feasibility of combining dialysis fluid-enhanced recipient CT, donor kidney segmentation, virtual fit simulation, and true-scale 3D-printed models for preoperative planning in pediatric kidney transplantation. The method provided patient-specific anatomical information on anticipated graft accommodation and supported surgical planning in small recipients receiving adult living-donor kidneys. Although no quantitative clearance thresholds were defined, the approach may complement conventional donor-recipient size assessment and surgeon judgment. Further prospective studies are needed to validate objective spatial metrics, assess clinical impact, and explore broader applications in recipients with complex anatomy or other transplantation settings.

## Data Availability

The raw data supporting the conclusions of this article will be made available by the authors, without undue reservation.
